# Patient characteristics and predictors of completion in residential treatment for substance use disorders

**DOI:** 10.1192/pb.bp.114.047639

**Published:** 2015-10

**Authors:** Giles Newton-Howes, James Stanley

**Affiliations:** 1 Otago University, Wellington, New Zealand

## Abstract

**Aims and method** To identify the patient characteristics and rates of retention in a residential rehabilitation drug and alcohol service (Springhill) based on an eclectic model of care. Patients were assessed using the Alcohol and Drug Outcome Measure (ADOM), a brief tool designed for the New Zealand setting. We looked at correlations between demographic, social and drug use parameters. Logistic regression assessed the relative impact of each variable on completion.

**Results** The 183 patients who completed the data collection did not differ from 47 non-completers by demographic data; 62.2% of patients completed the programme, with equal number of men and women. One in five participants was Maori, the indigenous minority. Alcohol (51.9%) was the commonest drug of misuse, with methamphetamine (16.4%) and cannabis (14.2%) also significant. Completers were more likely to be Maori, have conflict with family and housing problems, although the last became non-significant in logistic regression.

**Clinical implications** Retention rates are higher in Springhill than in comparable programmes. Ethnicity and family conflict predict completion, although the reasons for this are unclear. ADOM is an effective tool that can be used in a clinical setting to enable analysis of service provision.

Substance use disorders (SUDs) are relapsing and remitting disorders that affect a significant proportion of the community. One-year prevalence rates of alcohol use disorders range from 3.5%^1^ to 8.7%,^2^ with lifetime rates for SUDs reaching 12.3%.^3^ The commonest age of onset for alcohol and drug problems is early adulthood,^4,5^ although the diagnosis may not be stable at this point.^6^ Not only are SUDs common, they cause significant social morbidity,^1^ physical morbidity^7^ and mortality.^8^ The reasons for ongoing use in the face of such difficulties is unclear, however, this may be due to the compulsive nature of substance use.^9^ Despite this, recovery from alcohol use is an achievable goal,^10^ hastened by effective management strategies.

A wide range of treatment options are used clinically, adopting both harm reduction^11^ and abstinence^12^ models. The latter is often the preferred choice, yet the success of these treatments is well known to be poor.^13^ The most intensive intervention for addiction is residential rehabilitation,^14^ used in most countries and considered integral by almost half addictions clinicians.^15^ Drop-out remains a major difficulty for residential programmes^16,17^ and improved retention is related to improved outcomes.^18^ Patient factors such as family involvement and employment improve retention,^19^ as do interpersonal factors such as the therapeutic alliance.^20^ A number of programme factors may also be important, including the ratio of counsellors to patients and a structured programme of therapy, as well as a balance between contributing to the community, specific addictions work and free time.^21^ Psychopathology has not been found to be correlated with outcome^22,23^ and this is interpreted as support for residential treatment for patients with drug use disorders and coexisting mental disorder.^24,25^

Developing routine assessment of residential interventions enables both description of patients presenting to these services and analysis of outcome. Although previous research is a useful guide, it may not be generalisable. Country and culture are two factors that may have an impact on alcohol and drug use and its management.^26,27^ Similarly, the attitudes and beliefs of addictions clinicians are of significance in deciding who is referred for residential treatment.^28^ Differences in culture,^29^ policy,^30^ funding and politics^31^ shape service provision and necessitate country-specific research. They also point to the need for country-specific, ideally easy to implement, research tools that have the capacity to translate into routine clinical practice. Only one New Zealand study has examined residential interventions,^23^ despite the importance of understanding this costly intervention and for whom it is most appropriate. This study supported a ‘non-discriminating approach’ to referral but did not use New-Zealand-specific measures and assessed a long-term treatment setting. Replication and assessment in short-duration residential treatment settings is important to both compare outcomes and examine possible differences.

For these reasons, we investigated the outcomes in a short-duration (8-week), eclectic residential programme using a locally designed and validated tool and report on factors that were associated with programme completion.

## Method

In total, 230 clients consecutively admitted to the Springhill residential rehabilitation programme were included in this study. These clients were admitted during 2011/2012. All completed standard clinical documentation on admission to Springhill and in addition were asked to complete an Alcohol and drug Outcome Measure (ADOM) on admission and discharge. The research was approved by the Southern Health and Disability Ethics Committee and the local district health board.

### The programme

Springhill runs an 8-week residential rehabilitation programme for all substance use disorders. Up to 15 patients can be admitted at any one time. Patients undergo managed withdrawal with their referring teams before admission if this is considered necessary, particularly from high-dose alcohol. It is unique in New Zealand insofar as the clinical programme is delivered by clinicians from a district health board (a public health provider) but the facility is governed by a private trust. All clinicians are registered health practitioners with clinical expertise in addiction treatment. The programme has input from a substance misuse psychiatrist and general practitioner with a special interest in addictions providing medical oversight. The programme is abstinence based, including abstinence from tobacco, and draws on principles from the 12-step programme, cognitive-behavioural therapy, family systems theory and some elements of a therapeutic community. In this way it is similar to residential programmes elsewhere^32^ and would best be considered as eclectic. Each week is structured around facilitated large group sessions with all clients, supplemented by individual and family work. Exercise and involvement in the running of the facility are part of the programme. Each client has an individual therapist to work with alongside the group and all clients are reviewed medically and have access to psychiatric review if necessary. At the conclusion of the programme clients are referred back to their community addictions providers.

### Client characteristics

Patients are accepted primarily from secondary addictions services in the community when this intervention has been ineffective as judged by their community clinician. There are few specific exclusion criteria and referrals are assessed on a case by case basis. Referrals are received from the lower North Island of New Zealand and include both rural and urban settings. Programme entry requires that there is a service to refer the client back to on completion. In the case of abrupt discharge each client has a person whom they have agreed to be contacted, with the referring service contacted the following day. If there are clinical concerns, a psychiatric crisis team is available to undertake assessment as deemed necessary. A clinical diagnosis of alcohol or drug dependence is a requisite for consideration for admission. Judicial direction is a specific exclusion criterion (i.e. clients cannot be legally directed into treatment at Springhill).

### The ADOM tool

The ADOM is a brief tool to collect outcome data and measure improvements after intervention in substance dependence, specifically in a New Zealand setting. Part A of the tool is validated^24^ and part B is similar to other tools used to collect information on social support and context, in line with other addictions rating instruments.^33^ Part A focuses on drug use, particularly number of days' use in the past 4 weeks and total use of alcohol in units, this being the most prevalent drug of misuse in New Zealand. Tobacco use is measured as average number of cigarettes a day. Part B asks clients to rate the frequency of problems in multiple social domains as a result of drug dependence, again in the past 4 weeks. These include problems related to physical health, mental health, conflict in interpersonal relationships, difficulties with work or other structured activity, housing and criminal activity. These are self-reported on a 1–5 Likert scale. A score of 1 represents no difficulty in the social variable measured, whereas a score of 5 represents daily or almost daily problems. These variables were measured for the 4 weeks preceding admission during engagement with community alcohol and drug services. This allowed for a measure of current social problems and can allow for examination of changes on these scales over time. The advantage of a self-report measure is its acceptability to patients and the reporting of perceived difficulty as assessed by the client.

### Data collection and analysis

At admission basic demographic information was collected from the patient and the ADOM tool was completed by the patient with their admitting therapist. At discharge, all patients completed discharge paperwork with their discharge therapist and the ADOM tool was again administered at this point. If clients were discharged outside of working hours they were contacted the following day to complete the ADOM if this was possible. All analysis was carried out in SPSS 19 for Windows and undertaken by one of the authors (J.S.).

The primary goal of this analysis was to identify what, if any, factors were associated with retention in the programme to 8 weeks or attrition over the 8 weeks. The purpose of this analysis is to provide services with information about clients who are at greater likelihood of succeeding to completion. All demographic and ADOM data used in this analysis are based on the measures collected at admission.

Descriptive statistics on sociodemographic and basic clinical characteristics (as collected at admission) are reported as means, standard deviations and proportions as appropriate. Logistic regression analysis was used to examine unadjusted relationships between each of the potential predictor variables and the outcome of completing the programme (defined as 8 weeks' participation in the residential programme). The predictor list set was developed *a priori*.

To consider the potential impact of confounding on the modelled estimates, subsequent logistic regression analysis looked at adjusted estimates for programme completion. Adjusted results are presented in four blocks. First, sociodemographic variables (age, gender and ethnicity) were entered into the model. Block two consisted of adding the primary drug of dependence as identified by the patients themselves (simplified to alcohol, cannabis, amphetamine and other drugs (e.g. opioids) and no drug specified). The third block included adding physical and psychological health variables and finally, social variables including work, paid employment, housing and crime. The model was developed to identify for whom completion was most likely as a proxy marker for positive long-term outcomes.

## Results

### Patient demographic data

During the study period 230 patients were admitted to Springhill, of whom 183 (80%) completed ADOM data on admission and discharge. The 47 patients for whom complete data were not available did not differ from the included patient data-set on basic demographic data. Overall, 143 patients (62% of all admitted patients) completed the 8-week programme (including 2 patients who stayed 9 weeks and 1 patient who stayed 10 weeks). For those included in the analysis the average age was 37 years with equal numbers of men and women. The majority of clients (74%) were White, 21% were Maori and the remainder identified with other ethnic backgrounds. At the time of admission 33% of clients had a partner, although 1 in 5 declined to answer this question. These variables are similar to those of clients not included in further analysis and similar to the demographic of the region (Table 1).

**Table 1:** Demographic and drug use of the client group

Client status (at admission)	Analysed data (*n* = 183)	Missing data (*n* = 47)	Total (*n* = 230)	*N* valid by question
Age, years: mean (s.d.)	37.4 (11)	36.2 (11.5)	37.1 (11.1)	230 (100%)

Gender				
Male	96 (52.5%)	20 (58.8%)	116 (53.5%)	217 (94.3%)
Female	87 (47.5%)	14 (41.2%)	101 (46.5%)	

Ethnicity				
Maori	39 (21.3%)	3 (23.1%)	42 (21.4%)	196 (85.2%)
White	136 (74.3%)	9 (69.2%)	145 (74%)	
Other specified	8 (4.4%)	1 (7.7%)	9 (4.6%)	

Relationship status				
Partner	51 (32%)	8 (36%)	59 (33%)	181 (79%)
No partner	108 (68%)	14 (64%)	122 (67%)	

Main drug at admission				
Alcohol	95 (51.9%)	9 (33.3%)	104 (49.5%)	210 (91.3%)
Cannabis	26 (14.2%)	7 (25.9%)	33 (15.7%)	
Amphetamine, other^a^	30 (16.4%)	5 (18.5%)	35 (16.7%)	
None selected	32 (17.5%)	6 (22.2%)	38 (18.1%)	

Substance use in past 4 weeks by main drug of dependence,^b^ days: mean (s.d.)				
Alcohol (*n* = 104)	13.2 (9.5)	11.1 (4.9)	13.1 (9.2)	95 (91.3%)
Cannabis (*n* = 33)	16.4 (11.8)	12.7 (12.8)	15.6 (11.9)	26 (78.8%)
Amphetamines^c^ (*n* = 27)	4.2 (7.6)	9.3 (12.7)	4.9 (8.4)	23 (85.2%)
Cigarettes (*n* = 230)	12.4 (12.8)	13.5 (11.7)	12.5 (12.6)	183 (79.6%)

a. A total of 8 clients reported using other substances outside the main categories.

b. Days' use represents the number of days' use of the substance identified as the primary drug of dependence by the client. The exception is mean number of days nicotine used; this is for the whole sample.

c. Amphetamine usage among those with amphetamine as main drug at admission.

### Drug use variables

The commonest drug of dependence in the sample was alcohol with 51.9% of clients identifying this as their most problematic drug. Amphetamine, primarily methamphetamine (16.4%), and cannabis (14.2%) were the other significant primary drugs of misuse. Thirty-two patients could not identify a primary drug of misuse, having problems associated with multiple drug use. Eight patients identified sedatives, opioids or other drugs as their primary drug of dependence with no more than three in each group. Twelve patients used the intravenous route for drug administration in the 4 weeks before admission, with 3 patients sharing needles.

Patients with primary alcohol dependence drank on average 14 units a day in the month before admission, with alcohol consumed for 13 of the past 28 days. Those with cannabis dependence smoked on 16 of the past 28 days on average. The 27 patients with amphetamine dependence used on 4 of the 28 days before admission. All groups were engaged in community alcohol and drug out-patient programmes prior to their admission. Tobacco smoking was common in this group despite the admission criteria to Springhill identifying it as a smoke-free rehabilitation facility, with the expectation of discharge if smoking occurred during the admission.

### Social variables

A quarter of clients stated their drug use led to daily physical health problems, with almost two-thirds identifying physical health problems occurring at least weekly and directly related to their drug use. Mental health problems occurred on a daily basis for one in seven patients, with three-quarters identifying mental health difficulties related to the use of drugs of dependence. Social difficulties were also common: 20% of patients recorded daily conflict with family or friends and almost two-thirds recognised conflict at least weekly. Recreational and work were similarly compromised, with 83% and 67% of clients respectively identifying problems at some point. Housing was a daily problem for three-quarters of clients. Despite the specific exclusion of referrals from the judicial systems, more than 90% of clients identified crime (other than the use of illicit substances) as related to their drug use, with more than half stating this was an almost daily problem.

### Univariate predictors of programme completion

Sociodemographic, clinical and social variables were examined as univariate predictors of programme completion using logistic regression (Table 2). Identifying as Maori, conflict with family and problems with housing were associated with increased rate of retention. Only ethnicity had an odds ratio greater than 2. Drug of dependence was not associated with programme completion and no association was found between coexisting mental health problems and programme completion.

**Table 2:** Unadjusted odds ratios for completion of the 8-week residential rehabilitation programme among respondents with complete data (*n* = 183)

Factor	Odds ratio (95% CI)	*P*
Age (analysed in 5-year blocks)	1.13 (0.98, 1.29)	0.10

Gender		
Male	0.97 (0.53, 1.77)	0.92
Female	1 (reference)	

Ethnicity		
Maori	2.31 (1.02, 5.23)	0.04
White/other	1 (reference)	

Primary drug of dependence		
Alcohol	1 (reference)	0.74
Cannabis	0.69 (0.29, 1.68)	
Amphetamine/other	0.66 (0.29, 1.54)	
None selected	0.85 (0.37, 1.95)	

Physical health problems^a^	1.05 (0.87, 1.27)	0.64

Mental health difficulties^a^	1.02 (0.83, 1.24)	0.97

Conflict with family^a^	1.30 (1.04, 1.62)	0.01

Social role difficulties^a^	1.17 (0.96, 1.42)	0.08

Employment problems^a^	1.10 (0.92, 1.32)	0.24

Housing problems^a^	1.47 (1.10, 1.96)	0.03

Crime^a^	1.11 (0.87, 1.41)	0.40

a. Odds ratios are increase in odds of completion per one unit in change in the scale.

### Regression modelling of programme completion

Logistic regression was carried out using a four-block, sequential adjustment method. Regression estimates (presented as odds ratios and 95% confidence intervals) are presented in Table 3. Model A included age, gender and ethnicity as predictors of treatment, model B added in the primary drug of dependence, while model C added scores measuring physical health, mental health and levels of conflict with family. The fully adjusted model (model D) added variables on social role, employment, housing and crime.

**Table 3:** Logistic regression of factors associated with completion of residential treatment programme among clients with complete data (n = 183)

	Model A		Model B		Model C		Model D	
Response	Odds ratio (95% CI)	*P*	Odds ratio (95% CI)	*P*	Odds ratio (95% CI)	*P*	Odds ratio (95% CI)	*P*
Age (per 5 years)	1.14 (0.99, 1.32)	0.07	1.12 (0.96, 1.31)	0.14	1.1 (0.94, 1.29)	0.231	1.08 (0.91, 1.27)	0.38

Gender								
Male	1.01 (0.55, 1.87)	0.97	1.03 (0.56, 1.92)	0.92	0.81 (0.41, 1.61)	0.55	0.80 (0.39, 1.63)	0.54
Female	1 (reference)		1 (reference)		1 (reference)		1 (reference)	

Ethnicity								
Maori	2.48 (1.08, 5.66)	0.03	2.57 (1.10, 5.98)	0.030	2.83 (1.18, 6.75)	0.02	2.82 (1.15, 6.92)	0.02
White/other	1 (reference)		1 (reference)		1 (reference)		1 (reference)	

Primary drug of dependence								
Alcohol			1 (reference)	0.93	1 (reference)	0.92	1 (reference)	0.95
Cannabis			0.72 (0.27, 1.89)		0.70 (0.26, 1.93)		0.74 (0.26, 2.08)	
Amphetamine/other			0.88 (0.35, 2.17)		0.91 (0.36, 2.28)		0.94 (0.36, 2.47)	
None selected			0.93 (0.40, 2.19)		0.88 (0.36, 2.13)		0.88 (0.36, 2.15)	

Physical health problems^a^					1.09 (0.86, 1.39)	0.46	1.11 (0.86, 1.42)	0.434

Mental health difficulties^a^					0.86 (0.66, 1.12)	0.26	0.85 (0.64, 1.12)	0.248

Conflict with family^a^					1.47 (1.12, 1.93)	0.01	1.44 (1.03, 2.00)	0.031

Social role difficulties^a^							1.01 (0.74, 1.38)	0.96

Employment problems^a^							1.13 (0.92, 1.39)	0.25

Housing problems^a^							1.35 (0.97, 1.89)	0.078

Crime^a^							1.00 (0.73, 1.36)	1

a. Odds ratios are increase in odds of completion per one unit in change in the scale.

In the fully adjusted regression model (model D), only increasing conflict with family and ethnicity (being Maori) significantly predicted completion of the residential rehabilitation programme. Of all variables, being Maori more than doubled the odds of programme completion. Drug use was not predictive of completion. Notably, there was significant use in the 28 days prior to admission and no medical detoxification offered. This suggests clients did not leave rehabilitation because of withdrawal phenomena. Housing problems were not significantly associated with programme completion in the full model, but the magnitude of the observed odds ratio and confidence interval (OR = 1.35, 95% CI 0.97, 1.89) suggest that such problems might also be associated with increased rates of programme completion. The modelling was reviewed using ordinal regression with weeks completed from 1 to 8 as the step-wise variable. This statistical model was largely the same as the linear regression, with the only alteration being the somewhat increased association between criminal activity and completion. Using ordinal modelling criminal activity significantly predicted greater length of stay (regression model available on request).

## Discussion

The current research uses a specific tool, ADOM, to assess patient characteristics and potential indicators of programme completion within a residential rehabilitation setting in New Zealand. The ADOM tool was acceptable to patients with high completion rates, despite more than a third of patients leaving the service early. It was easily intercalated into routine clinical care and specific researchers were not used to collect this information, indicating its capacity for translation for routine use (see www.matuaraki.org.nz/supporting-workforce/adom). The potential for use throughout New Zealand would allow for in-depth understanding of addiction service use and potentially prediction of service matching.

Patients entering rehabilitation report high ongoing use of alcohol and other drugs, despite being engaged in community treatment programmes prior to entry. This implies difficulties for some patients with SUDs despite community intervention and the need to develop and deliver alternative interventions. Alcohol remains the most significant drug of dependence in those referred for residential support, although methamphetamine use is common. Low opioid use and low injecting rates may reflect the effectiveness of opioid substitution regimes.^34^ The need for a drug-free environment is well recognised as a component in recovery for some patients and this is likely to remain the case. Completion rates of greater than 60% are high, with other programmes reporting rates as low as 16%,^35^ and likely reflect a physical, social and therapeutic environment that is acceptable to patients. The lack of association between specific alcohol or drug use and completion demonstrates the ability to remain in a residential setting despite the varying biological impact of different drug classes and argues for a generic approach to treatment rather than one that is drug specific.

Significant morbidity is reported in physical, psychological and social domains by the patients in this study.These problems are directly related to drug use and small changes in use are likely to be associated with significant benefits to health, relationships and well-being. Previous economic analysis indicates major benefits associated with effective addictions intervention also.^36^ The relative failure of community intervention for this cohort argues in favour of residential intervention, particularly if retention is high, and implies improved prognosis. This is the case for Springhill and may relate to positive longer-term outcomes.^37,38^ Follow-up studies will enable further examination of longer-term benefits and overcome the limitation of using completion as a proxy marker for improved prognosis.

Identifying who is likely to benefit most from residential treatment allows for a more targeted approach to management. Prior research has recommended a ‘non-discriminatory approach to referral’ and no clear indicators are apparent in the current literature base. Using regression analyses to consider the impact of several factors likely to alter treatment completion, we are able to show that Maori, the indigenous minority in New Zealand, and those with conflict in the home are more likely to complete the programme. The programme includes the capacity for patients to engage with a cultural assessment but does not include individual or group activities that are specifically culturally oriented. Previous research identifies greater social morbidity in Maori in an out-patient addictions setting^39^ and greater satisfaction with a culturally specific service. Cultural factors have been a point of focus in policy debate about the provision of services,^29^ with some advocating for a culturally appropriate approach research methodology frame, although support (and the application) of this is very limited. The current findings suggest Maori manage well in a generic eclectic setting. This does not indicate a generic service is likely to outperform a culturally specific service; rather, Maori are more likely than clients of other ethnicities to complete this programme. Ethnicity is not a proxy marker for social disadvantage as measured by social role difficulties, employment, housing problems and crime in this study as the linear regression of model D elucidates. Understanding the impact of homelessness in dependence is complex,^40^ although the parsimonious explanation of having basic needs met does not preclude the potential for recovery and may be an important component of successful recovery.

The findings of this research are circumscribed by the limitations related to a naturalistic cohort design. Not all questions were completed by all patients and this leads to a weakening in the analysis, however, the advantage of developing the data collection as part of routine care confirms its utility and capacity for translation from research to routine use. The benefits of using a country-specific tool may counterbalance the restrictions of its limited use. The outcome measure of programme completion is useful,^37,41^ however, long-term abstinence is a more powerful outcome. Follow-up studies are necessary to assess this.

This study highlights the acceptability for some of a residential setting to address their substance dependence. Significant pre-entry drug use, despite community care, does not prevent engagement and abstinence-based programme completion. Maori and homeless patients engage well and these factors predict retention. ADOM is a potentially valuable tool for monitoring outcome from a residential setting and has wider potential use in New Zealand.
